# Mindreading and metacognition patterns in patients with borderline personality disorder: experimental study

**DOI:** 10.1007/s00406-020-01227-7

**Published:** 2021-01-18

**Authors:** Tomasz Cyrkot, Remigiusz Szczepanowski, Kamila Jankowiak-Siuda, Łukasz Gawęda, Ewelina Cichoń

**Affiliations:** 1 College of Psychological Sciences, Faculty of Education, University of Lower Silesia, Wroclaw, Poland; 2 Behavioral Neuroscience Lab, SWPS University of Social Sciences and Humanities, Warsaw, Poland; 3 Experimental Psychopathology Lab, Institute of Psychology, Polish Academy of Sciences, Warsaw, Poland; 4 WSB University in Toruń, Toruń, Poland

**Keywords:** Mindreading, Metacognition, Borderline personality disorder, Post decision wagering

## Abstract

Current psychopathology attempts to understand personality disorders in relation to deficits in higher cognition such as mindreading and metacognition. Deficits in mindreading are usually related to limitations in or a complete lack of the capacity to understand and attribute mental states to others, while impairments in metacognition concern dysfunctional control and monitoring of one’s own processes. The present study investigated dysfunctional higher cognition in the population of patients with borderline personality disorder (BPD) by analyzing the accuracy of metacognitive judgments in a mindreading task [reading the mind in the eyes Test (RMET)] and a subsequent metacognitive task based on self-report scales: a confidence rating scale (CR) versus a post-decision wagering scale (PDW). It turned out that people from the BPD group scored lower in the RMET. However, both groups had the same levels of confidence on the PDW scale when giving incorrect answers in the RMET test. As initially hypothesized, individuals with BPD overestimated their confidence in incorrect answers, regardless of the type of metacognitive scales used. The present findings indicate that BPD individuals show dysfunctional patterns between instances of mindreading and metacognition.

## Introduction

Borderline personality disorder (BPD) is a severe mental condition characterized by a pervasive pattern of marked impulsivity and instability of affect, self-image and interpersonal relationships [1]. Furthermore, a constant feeling of abandonment and emptiness and frequent impulsive self-harm and behaviors lead to severe social dysfunctions in individuals diagnosed with BPD [1]. In line with this view it has been suggested that BPD symptoms are underpinned by deficits in the emotion perception and interpretation of social signals [2–4]. The previous studies on abnormalities in BPD have showed that misinterpretation of mental states of others and emotional instability are causes of extreme reactions and dysfunctional social contacts [5]. Dziobek and co-workers [6] also indicate an increased level of emotional arousal, which is part of BPD symptomatology, as a possible cause of difficulties in differentiating emotional states. Thus, emotional dysregulation of BPD meant as a combination of high sensitivity to emotional stimuli, elevated stated of arousal, and a low return to emotional baseline, shaped by social context, can impair the ability to read other people's mental states [7]. The consistent body of behavioral and neuropsychiatric research has shown that difficulties in social cognition are one of the central problems in BPD [8–11]; the literature also supports the idea that the disturbed relationships of BPD patients may be a more specific symptom of psychopathology than the emotional instability that is displayed by those with this disorder [4]. It is important to mention that biases in social cognition are specific symptoms for another mental disorders such as depression [12], social anxiety disorders [13] or eating disorders [14].

Some studies have shown that people with BPD may have difficulty in recognizing the facial expressions of others [15, 16]. Patients with BPD have higher error rates for recognition of emotional expressive faces compared to healthy controls, though they display higher accuracy in detecting fearful faces [15]. However, other results have revealed that BPD patients are less accurate than control participants in emotion recognition, particularly when discriminating negative emotions, but they are not impaired in the recognition of happy facial expressions [16]. There are also studies showing no alterations or deficits in facial or prosodic emotion recognition in BPD population (e.g. Minzenberg et al. [17]). Some other studies suggest that disruption of facial recognition expressions does not affect multimodal emotion recognition ability as information channels other than facial expressions are used to recognize social signals [18]. There are also reports indicating that BPD patients show the intact capacity to understand behavior of others just like healthy individuals [19, 20].

It is important to mention that the social cognition deficits observed in BPD are not limited to the emotion recognition. As several other studies suggest, there are also impairments in Theory of Mind (ToM) [21] in terms of understanding the mental states, emotions, desires, beliefs or intentions of others [22]. Indeed, ToM deficits may result in misunderstanding others’ mental states and intentions and thus constitute a major source of interpersonal difficulties across a wide range of mental disorders, including BPD [21]. These effects were studied by the reading the mind in the eye test (RMET) measurement, originally developed by Baron-Cohen and coworkers [23]. In particular, RMET assesses the ability to recognize the mental states of other people based on information gathered from facial expressions [23]. Some studies using RMET have indicated that patients with BPD do not differ from healthy control groups, [24, 25] but these findings were not replicated in studies by Fertuck et al. and Frick et al. [26, 27] whose BPD groups performed better than healthy controls in the positive as well as negative RMET conditions. It turned out in both studies that there were no group differences, only in the neutral RMET condition. In another experiment which studied a non-clinical BPD group, [28] the participants performance was better for negative RMET but not for positive valances. Other findings on social cognition deficits in borderline disorders revealed poor mentalizing abilities in RMET in terms of recognizing photographs of the eye region with negative and positive valences [29] but this was only true in the case of positive valence in the study by Petersen [30].

The abovementioned reports collectively suggest that there are inconsistencies in the findings on the accuracy of mental state recognition among patients with BPD [21, 31]. At the same time, several studies suggest that BPD individuals may have dysfunctional meta-knowledge of the recognition of emotional cues and are thus unsure of their decisions. This hypothesis may be investigated by combining assessment of emotion recognition with the relevant measure of metacognitive confidence. For instance, Thome et al. [32] investigated dysfunctional patterns between metacognition and emotion recognition by inspecting how BPD patients differed in confidence while assessing faces with emotion blends (happy, angry and neutral facial expressions) presented on the computer screen. In particular, it was shown that people with BPD reported lower confidence than healthy participants while assessing the intensity of emotional expression of happy faces; both groups did not differ in confidence judgments on recognizing the intensity of angry faces. Kaletsch and co-workers [33] investigated how borderline personality disorder may alter the relationship between emotion perception of body movement and confidence judgments. Findings presented in the work by Kaletsch and co-workers [33] implicated that BPD patients had no biased perception in either a positive or negative direction of perceived emotional scenes, although they were less confident in perceiving emotional scenes with low intensity [33]. There is only one study by Schilling and co-workers [25], which indicated that BPD individuals produce similar results to healthy persons in the RMET task, although their pattern of metacognition responses is different because they tend to be overconfident in incorrect answers.

Taken together, these reports may lead to the observation that there are inconclusive results in BPD individuals in terms of their social cognition and metacognition. Overconfidence in incorrect responses should be treated as an important factor that reflects the social functioning of BPD individuals [34] because lack of correctness in task execution and abnormal confidence might be understood as specific symptoms of BPD. In particular, a patient’s inability to correct their own interpretations despite perceptual errors in a social context seems to be of key importance when solving conflicting social situations [35]. In our opinion, the current research is insufficient to obtain an in-depth understanding of the relationship between the metacognition and mindreading mechanisms in BPD. To fill this gap, the present study applied other measures to investigate metacognitive mechanisms based on economic cues [36, 37]. Namely, the important research question is whether BPD individuals can display the correct, cautious metacognitive strategy in this kind of economic task. In turn, the results of Schilling and co-workers [25] indicate deficits in metacognition in BPD disorders, but only with typical measures of confidence ratings. Thus, our study goes one step further and attempts to observe whether similar abnormal mechanisms are used when evaluating confidence using economic categorizing. This method of evaluating metacognition with small monetary stakes (so-called post-decision wagering) triggers loss-aversion mechanisms in healthy participants, thus activating avoidance strategies to avoid losses [36, 38]. It is likely that individuals with BPD trigger such an evolutionary loss-aversion mechanism at some point [39], but its adaptive impact of avoidance on behavioral functioning may be aggravated when undertaking decision-making tasks due to the fact that abnormal metacognitive mechanisms of monitoring and control are engaged by this population.

Therefore, in the present study we used RMET and two types of confidence scales to assess ToM abilities and metacognition, respectively: a typical confidence rating scale (CR) [40–43] and the *post-*decision wagering scale (PDW) [44, 45] and we hypothesized that individuals with BPD would exhibit lower performance in the eye test (RMET) than healthy controls. As regards the patients’ evaluations of metacognition, in comparison to healthy controls, it was assumed that the BPD population would overestimate confidence in both correct and incorrect answers. In addition, it was expected that the overestimation effect in metacognitive confidence would be significantly higher when using post-decision wagering.

## Methods

### Participants

The study examined 33 patients with a diagnosis of borderline personality disorder (BPD) (32 female;1 male) and 33 healthy participants (HP) (31 female; 2 male). Patients were recruited from the psychiatric ward of the ‘MARIAMED’ Centre of Psychiatry and Psychology in Lubin, Poland. The control group was recruited from among students of psychology from SWPS University of Social Sciences and Humanities, Faculty in Wroclaw; all participants completed informed consent forms. A structured Clinical Interview for DMS-IV Axis II (SCID-II) [46] was used to diagnose BPD patients. No features of addiction or psychotic symptoms were revealed in the BPD patients.

In addition, the BPD patients were examined for concomitant disorders using the Mini International Neuropsychiatric Interview (MINI). We also used MINI in a control group in which there was no evidence of mental disorders. Given the results of the MINI inventory and previous diagnosis, it was established that 7 patients (21%) met the criteria for mood disorders (depression); 11 patients (33%) met the criteria for anxiety disorders; 13 patients met the criteria for adaptation disorders (39%); 1 patient met the criteria for eating disorders (3%); 1 patient met the criteria for alcohol dependency syndrome (3%). The detailed characteristics of patient symptoms according to the DSM-V criteria is set out in Table 1.

**Table 1:** BPD symptoms according to DSM-V and percentage of patients displaying particular diagnostic criteria in the structured clinical interview (SCID-II)

Criteria for BPD (DSM-V)	% of patient
Efforts to avoid real or imagined abandonment	66%
Unstable and intense interpersonal relationships	69
Unstable self-image or sense of self	53%
Impulsivity in at least two areas that are potentially self-damaging	72%
Recurrent suicidal behavior, gestures, suicide threats, or self-mutilating behavior	51%
Affective instability due to a marked reactivity of mood	90
Chronic feelings of emptiness	84%
Inappropriate, intense anger or difficulty controlling anger	52%
Transient, stress-related paranoid ideation or severe dissociative symptoms	48%

### Reading the mind in the eyes test

The present study used a Polish version of the RMET [23, 47]. The RMET task consisted of 36 black-and-white photos of only the eye region of faces (18 males) expressing complex mental states. For each of the photos, four adjectives describing mental states were displayed near the corners of the photograph, of which only one was regarded as correct. The RMET task and metacognitive scales were presented on the computer screen using the Super Lab program. A green dot (not present in the original test) was added between the eyes in each photo; this dot was used as a fixation point to make sure that the facial image was more likely to be focused on by participants. The participants were asked, “what is the person in the photograph feeling or thinking?”. Then, the participant pointed and clicked with the computer mouse on one of the four possible adjectives describing mental states.

Based on valence classification, the percentage of correct answers was calculated for the RMET scale (36 items) and for the subscale scores: for positive valence (8 items: playful, fantasizing (2x), thoughtful, friendly, interested, flirtatious, confident); negative valence (12 items: upset, worried, regretful, accusing, doubtful, preoccupied, defiant, hostile, cautious, distrustful, nervous, suspicious); and neutral valence (16 items: desirous, insisting, uneasy, despondent, preoccupied, cautious, skeptical, anticipating, contemplative, decisive, tentative, pensive, interested, reflective, serious, concerned).

**Fig. 1:**
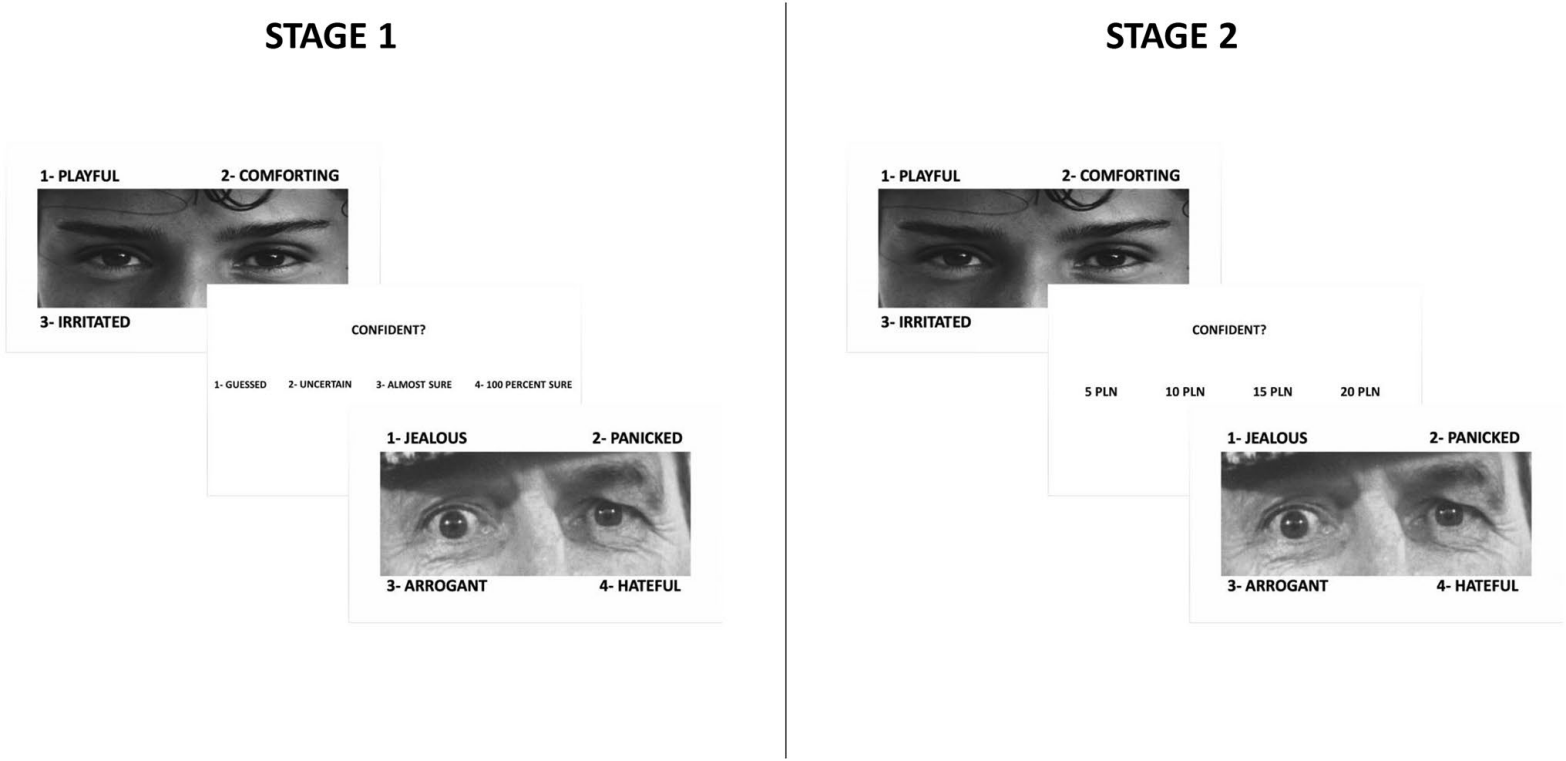
Experimental design and procedure. The facial image was presented on the screen; the green dot in the center encouraged participants to pay attention to the face. Then, participants had to choose one of the four possible expressions presented alongside the photo. Participants then assessed their confidence with metacognitive scales using confidence ratings (CR scale) or imaginary monetary wagers (PDW). The order the of measure of metacognition was randomized across the participants

Then, after presentation of each picture on the monitor, participants rated their confidence using two metacognition scales. We used two measures of metacognition: confidence rating (CR) and post-decision wagering (PDW). CR was used to assess confidence on the verbal scale (1: ‘a total lack of confidence’, 2: ‘uncertain’, 3: ‘almost sure’, 4: ‘absolutely sure’). The PDW scale measured confidence by asking participants to wager their recognition decision with imaginary money. Wagers of 5 PLN, 10 PLN, 15 PLN, 20 PLN (1 Euro equals approx. 4.29 PLN) were used to express confidence in given responses. The order of the administrating scales (CR vs. PDW) was randomized across participants. The time allowed to answer was not limited and response time was not measured. Participants performed the task twice (in two stages), responding in each stage on one of the two different confidence scales (CR vs. PDW). The order of the rating task was randomized across participants. The experimental procedure is presented in Fig. 1.

### Statistical analyses

The group differences in terms of performance in the RMET test and measures of confidence were examined with the analysis of variance (ANOVA) method following a mixed design with two within-subjects variables and a between-group variable. In the case of RMET performance, the analysis included the following variables: group variable (BPD vs. HP), scale type (CR vs. PDW), valence (negative vs. positive vs. neutral), and age as a control variable. The analysis of metacognitive measures took into account confidence responses that were collapsed into the following categories: group (BPD vs. HP), scale type (CR vs. PDW), valence (negative vs. positive vs. neutral), correctness of responses in the eye test (incorrect vs. correct responses), and age as the control variable. We also used another approach to analyze metacognition based on the KCI Knowledge Corruption Index (KCI) (see definition below). A three-way mixed-design ANOVA model considered the following factors: group variable (BPD vs. HP), measure type of metacognition (CR vs. PDW), valence (negative vs. positive vs. neutral), and the control variable (age).

The final analysis used another measure of metacognition: knowledge corruption index (KCI). This parameter was computed either for the PDW or CR scales and represented the proportion of failures in RMET responses followed by the highest confidence (“Totally certain”) responses [57]. Increased KCI values for incorrect answers suggest that individuals have false beliefs supported by strong confidence that their answers are correct. On the other hand, a low KCI for incorrect answers indicates that people are convinced that their incorrect answers are false (true beliefs).

## Results

### Performance in the eyes test

There were significant age differences between groups *t* (64) = −2.30, *p* < 0.05, yielding *M* = 32.15, SD = 6.88 for the BPD patients and *M* = 28.06, SD = 7.50 for the healthy controls. Therefore, the three-way mixed ANOVA analysis took the age factor (the covariant) into account to examine group differences. The analysis indicated the main effect of the diagnosis *F* (1, 63) = 10.976, *p* = 0.002, η^2^_partial_ = 0.148 on performance in the Eye-test. We found that BPD patients achieved significantly lower scores (*M* = 71.756; SD = 1.413; 95% CI = 68.933–74.580) in recognizing mental states in the RMET test than healthy subjects (*M* = 78.507; SD = 1.413; 95% CI = 75.683–81.330). The ANOVA revealed no significant main effect of RMET valence, *F* (2, 126) = 2.066, *p* > 0.05, η^2^_partial_ = 0.032, and no interaction effect of group and scale, *F* < 1; there was no significant interaction effect between the diagnosis, RMET valence and the metacognitive scale, *F* < *1*.

### Measure of metacognition

A four-way mixed ANOVA analysis (correctness x group x scale x valence) was used to investigate metacognition. The analysis took the co-variable (covariant) of age into account. The ANOVA showed the significant main effect of correctness on metacognition, *F* (1, 35) = 10.261, *p* = 0.003, *η*^*2*^_partial_ = 0.227, indicating that the level of confidence obtained for correct responses (M = 3.153; SD = 0.446; 95% CI = 3.035–3.272) was higher than for incorrect responses (M = 2.702; SD = 0.586; 95% CI = 2.586–2.819), *p* < 0.001.

**Fig. 2:**
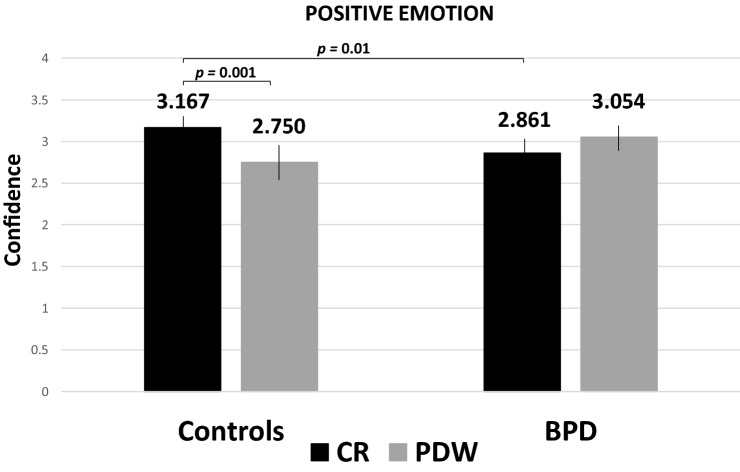
Post-hoc comparisons with Bonferroni correction: Mean confidence level for correct responses to positive emotion in HP and BPD groups in CR and PDW conditions

Furthermore, the ANOVA indicated a significant interaction between the group and scale, *F* (1, 35) = 11.004, *p* = 0.002, *η*^*2*^_partial_ = 0.239. However, there was a significant interaction effect between the emotional valence of the presented items and the measure of metacognition, *F* (1.609, 70) = 4.090, *p* = 0.030, η^2^_partial_ = 0.105, as well as a three-way interaction between the valence, the scale and the age variables, *F* (2, 70) = 3.444, *p* = 0.037, η^2^_partial_ = 0.090. The three-way interaction effect between the group and the valence and the measure of metacognition was approaching statistical significance, *F* (2, 70) = 2.940, *p* = 0.059, η^2^_partial_ = 0.077. We also observed the significant interaction between the valence, the scale and the correctness, *F* (2, 70) = 3.839, *p* = 0.026, η^2^_partial_ = 0.099; there was no interaction between the valance, the scale, the correctness, and age, *F* (2, 70) = 2.905, *p* = 0.061, η^2^_partial_ = 0.077; there was a trend, but no statistical significance for the four-way interaction between the valence, the scale, the correctness, and the group, *F* (2, 70) = 3.075, *p* = 0.052, η^2^_partial_ = 0.081. Then, post-hoc comparisons for the three-way interaction revealed the effects of the group on valence and metacognition for the CR scale and positive items (Bonferroni adjustments, *p* = 0.01 (Fig. 2).

**Fig. 3:**
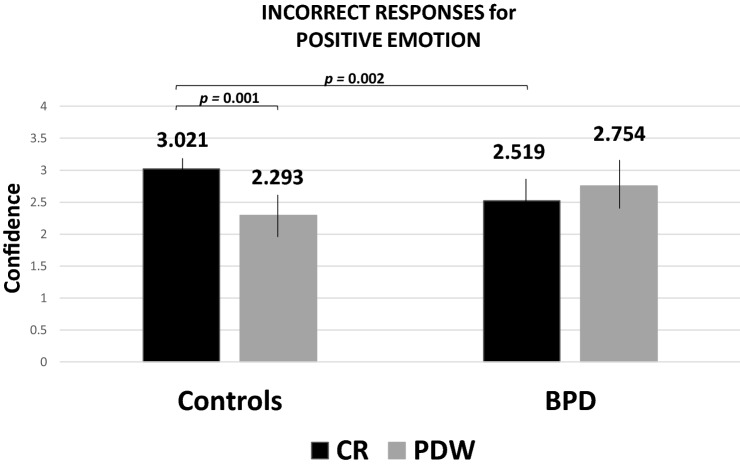
Post-hoc comparisons with Bonferroni correction: Mean confidence level for incorrect responses to positive emotion in HP and BPD groups in CR and PDW conditions

Furthermore, post-hoc analysis of the four-way interaction effects revealed significant differences between the groups for the CR scale (*p* = 0.002; Bonferroni corrections). In particular, BPD participants evaluated confidence in their incorrect responses to positive items significantly lower (*M* = 2.519; SD = 0.492; 95% CI = 2.300–2.739) when revealing confidence than healthy controls (*M* = 3.021; SD = 0.408; 95% CI = 2.813–3.228). In addition, for healthy participants there were significant differences in confidence for incorrect responses to items with positive valence (*p* < 0.001; Bonferroni adjustments), because confidence expressed with CR was higher than with PDW (Fig. 3).

Next, we took into account correctness as a criterion for producing confidence in the BPD and HP groups; there were significant differences between measures of metacognition for correct and incorrect responses. The analysis showed that application of the CR scale to healthy participants on items with negative valence (*p* = 0.017, Bonferroni corrections) led to higher confidence in correct answers (*M* = 3.106; SD = 0.455; 95% CI = 2.893–3.319) than in incorrect answers (*M* = 2.763; SD = 0.481; 95% CI = 2.515–3.011). Similarly, for healthy participants evaluations of confidence with the PDW scale indicated a higher level of confidence in correct answers (*M* = 2.994; SD = 0.491; 95% CI = 2.779–3.209) than in incorrect responses (*M* = 2.677; SD = 0.592; 95% CI = 2.378–2.976). For healthy participants, significant results (*p* = 0.022, Bonferroni corrections) were also obtained for positive emotions using the CR scale since the level of confidence in correct responses was higher (*M* = 3.313; SD = 0.349; 95% CI = 3.117–3.508) than in incorrect responses (*M* = 3.021; SD = 0.408; 95% CI = 2.813–3.228). As for the PDW measure of metacognition, we found that higher confidence was displayed for correct responses (*M* = 3.206, SD = 0.481; 95% CI = 2.950–3.462) than for incorrect responses (*M* = 2.293; SD = 0.730; 95% CI = 1.949–2.638), (*p* < 0.001, Bonferroni adjustments). The same confidence level patterns occurred for neutral emotions in the case of the CR measure, since the confidence levels for correct (*M* = 3.186; SD = 0.314; 95% CI = 3.004–3.369) and incorrect responses were different (*M* = 2.753; SD = 0.452; 95% CI = 2.513–2.993), *p* < 0.001. These patterns also occurred when the PDW was used: differences were found between correct (*M* = 3.031; SD = 0.362; 95% CI = 2.836–3.226) and incorrect answers (*M* = 2.529; SD = 0.548; 95% CI = 2.251–2.807) (all multiple comparisons with Bonferroni method, *p* < 0.001). This indicated that the HP group was more confident in correct answers than in incorrect ones when applying either CR or PDW to reveal metacognition.

The post-hoc analysis obtained for BPD patients showed the following. The results for mean confidence for negative emotions produced with the CR scale indicated no significant differences between the correct (*M* = 3.036; SD = 0.451; 95% CI = 2.811–3.261) and incorrect (*M* = 2.803; SD = 0.583; 95% CI = 2.541–3.066) answers (*p* > 0.05). However, there were differences on the PDW scale for the same valence (*p* = 0.036, Bonferroni correction), because confidence produced for correct (*M* = 3.177; SD = 0.452; 95% CI = 2.949–3.404) responses was evaluated higher than for incorrect responses (*M* = 2.872; SD = 0.741; 95% CI = 2.556–3.187). For items with positive valence, employment of CR for correct answers produced higher confidence (*M* = 3.202; SD = 0.486; 95% CI = 2.996–3.409) than for incorrect responses (*M* = 2.519 SD = 0.492; 95% CI = 2.300–2.739), *p* < 0.001. The same effect for confidence was obtained with the PDW scale, since the level of confidence for correct responses (*M* = 3.355; SD = 0.616; 95% CI = 3.084–3.626) was higher than for incorrect responses (*M* = 2.754; SD = 0.730; 95% CI = 2.390–3.118), (*p* = 0.004; Bonferroni adjustment). For neutral items, the CR scale indicated higher confidence for correct answers (*M* = 3.071; SD = 0.460; 95% CI = 2.878–3.264) than for incorrect responses (*M* = 2.630*; *SD = 0.579; 95% CI = 2.376–2.883), (*p* = 0.001). The same effect for neutral valence resulted from the PDW measure as the level of confidence in correct responses (*M* = 3.164; SD = 0.468; 95% CI = 2.958–3.369) was higher than in incorrect responses (*M* = 2.814; SD = 0.654; 95% CI = 2.520–3.108), (*p* = 0.011). Thus, on both scales BPD patients displayed a similar pattern of metacognition as healthy participants when correct and incorrect responses were evaluated.

### Knowledge corruption index (KCI) measure of confidence in incorrect responses

**Fig. 4:**
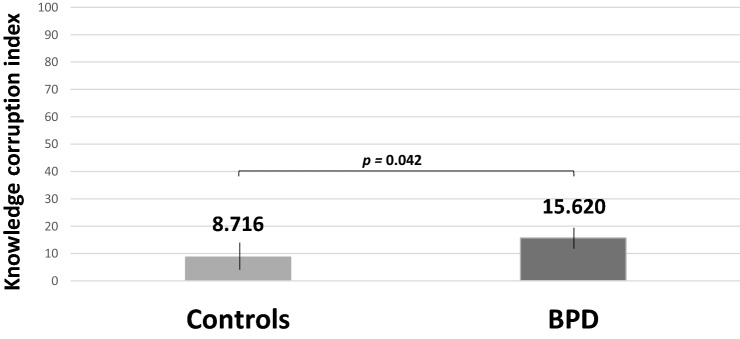
Post-hoc comparisons with Bonferroni correction: Mean KCI values for incorrect responses among HP and BPD groups

The KCI values were submitted to two-way mixed ANOVA analysis that considered the group variable (BPD vs. HP) and within-subject factors such as the valence of the item (negative vs. positive vs. neutral), the type of metacognitive measure (CR vs. PDW), as well as the age co-variant. The results indicated that there was a main effect of group, *F* (1, 42) = 4.380, *p* = 0.042, *η*^*2*^_partial_ = 0.094, since KCI value was *M* = 15.62 (SD = 2.285; 95% CI = 11.118–20.123) for BPD was higher than for healthy controls M = 8.716 (SD = 2.231; 95% CI = 4.105–13.326) (Fig. 4). There was a main effect of valence, *F* (2, 84) = 5.762, *p* = 0.005, *η*^*2*^_partial_ = 0.121. It turned out that healthy participants achieved lower KCI scores as compared to the BPD group thus indicating that the BPD group expressed a stronger tendency to overestimate confidence in incorrect answers as compared to controls. The post-hoc analysis of the effect of valance indicated the highest mean KCI in negative valence (*M* = 18.103, SD = 2.332; *95% CI* = 13.397–22.808), followed by neutral valence (*M* = 13.978, SD = 1.888; 95% CI = 10.168–17.787) and positive valence (*M* = 4.424, SD = 1.359; 95% CI = 1.681–7.167).

## Discussion

The present study investigated the recognition accuracy of mental states in the *Reading the mind in the eyes test* and metacognitive evaluations of such recognition between individuals with BPD symptoms and healthy populations. We found that BPD patients had lower accuracy in recognizing mental states as compared to the control group. Several clinical theories claim that BPD individuals perform worse in reading others’ mental states [48, 49]. Our finding of mentalizing abilities of BPD individuals based on the total RMET scores are consistent with the studies of Unoka et al. [16] and Anupama et al. [29] and with a non-clinical group of BPD adolescents [50]. However, we did not observe an effect of study group in RMET item valence (positive, negative, or neutral), whereas in Unoka and et al. [51] and Anupama et al. [29] this was present. In these studies, the BPD individuals recognized neutral and positive emotion in the RMET test to a significantly lower extent than control groups [51]; similar findings were found for the recognition of the negative and positive valence of mental states [29]. Our results suggest that the poor mindreading ability in the BPD group is related to the postulated poor mentalization [31, 52] and facial emotion recognition [53].

Nonetheless, the present results are inconsistent with the performance patterns found by Petersen and co-workers [30], who observed in the RMET that a BPD group achieved lower accuracy in recognizing positive mental states. Our results also show opposite patterns as compared to the studies of Fertuck et al. [26] and Frick et al. [27], suggesting that BPD patients generally score higher in the RMET test. These inconsistences might be attributable to the heterogeneity of BPD and its highly variable comorbidity profile [51]. Depression has been shown to have the effect on mindreading in one study on patients with BPD [26]. However, this has not been confirmed by other studies, such as Frick et al. [27], Preißler et al. [24] and Schilling et al. [25]. Additionally, inconsistences in RMET results could be related to the impulsiveness of BPD patients, who showed faster RMET responses than control groups in some studies, such as Frick et al. [27].

To the best of our knowledge, this is the first study that has investigated the relations between metacognition and mindreading in a BPD population by applying post-decision wagering based on monetary incentives. Research regarding differences in the assessment of metacognitive confidence has shown that monetary incentives can lead to more accurate estimation of confidence in responses related to emotional stimuli in the non-clinical population [54] and in memory tasks in the clinical population [37, 55]. Our study found no differences in PDW measures of metacognition between BPD patients and healthy participants. The comparisons of PDW measures showed that both groups equally applied loss-aversion strategies [38] when betting on their recognition accuracy. Interestingly—just as in the healthy population—this personality disorder does not prevent the use of *loss-aversion* strategies when making economic decisions.

When valence was taken into account, healthy participants assessed their confidence lower on the PDW scale than on the CR scale only in the case of positive valence. This suggests some type of avoidant decision-making strategy by which people naturally disclose an aversion to loss [56] and avoid unnecessary risk in economic decision-making in uncertain situations. Also, in the case of incorrect items with positive emotion, it was found that healthy individuals were more confident using the CR scale than those with BPD symptoms. As opposed to healthy controls both metacognitive scales did not differentiate confidence in BPD patients for incorrect responses. Therefore, loss aversion in BPD population does not seem to trigger a precautionary strategy that is often based on lower wager ratings when participants are not fully confident in their emotion recognition decisions [55].

It turns out that BPD patients expressed lower confidence on the CR scale than healthy controls, but only when evaluating confidence in positive valence items; the PDW scale did not provide similar results. This finding is consistent with the study by Thome et al. [32] who observed the reduced confidence in recognizing happy faces. Thome et al. [32] claimed that dysfunctional processing of positive items in borderline personality, driven by feelings of loneliness and expectation of social rejection (as indicated by correlation measures) alters experience of emotional intensity of stimuli and confidence during assessments [32]. In our study, we went a step further by providing evidence from analyses of three-way/four-way interactions suggesting that reduced confidence occurred only for incorrect answers on positive items. This, in turn, implies that BPD patients have more accurate metacognition (fewer levels of confidence) when wrongly recognized positive items.

It is important to note that there were no group differences in confidence in several RMET conditions, even though mindreading ability of BPD patients in our study was compromised. This could indicate at some point that borderline individuals did overestimate their metacognition in this study. Such clear pattern of overconfidence was observed in the study by Shilling et al. [25], since overall patients were more confident in the RMET responses, even though they had uncompromised mindreading ability. The analysis of the KCI index was our attempt to shed more light on this observed dysfunctional “overconfidence” when processing incorrect responses. When we looked at the KCI, which is the ratio of incorrect responses with a high level of certainty to the number of all responses with a high level of certainty [57, 58], a higher ratio was observed in patients with BPD. The higher KCI measures for BPD group indicates inaccurate knowledge leading patients to be convinced that their incorrect answers are true. Following these response patterns, one can conclude that overconfidence in errors may be more general processing mechanism of denial that prevents BPD patients from re-assessing available social cues adequately. Similar conclusions of the role of confidence in error in BPD population were drawn in studies of social cognition complemented by response confidence ratings [34, 59].

These results may also support the hypothesized dysfunctional regulation at the metacognition level in patients with a diagnosis of BPD if one thinks of metacognition as a system that supports monitoring, interpretation and evaluation in the regulation of current information processing [60, 61]. Therefore, abnormal metacognition may affect the self-regulation of behavior through the faulty processes of monitoring and control. In this view, metacognition dysfunctions that are characteristic of people with borderline personality disorder can develop and sustain impulsive behavior; as indicated in our study, this is due to the inability to change regulation strategy when using monetary wagers to evaluate social cues.

## Limitation

Our study has also limitations. First, the present work did not screen patients for current medication use in terms of doses and types of medication taken. Although several works on emotional recognition report no effect of medical use in BPD population (see for instance [62, 63]), further studies are needed to investigate effects of medication intake on metacognition and mindreading capacity in BPD patients. Second, because the present findings were related to three- and four-way interaction effects, the statistical inferences of confidence judgments effects might be debatable. For that reasons, our study employed the knowledge corruption index to resolve this inconsistency.
